# Neurosteroid allopregnanolone (3α,5α-THP) inhibits inflammatory signals induced by activated MyD88-dependent toll-like receptors

**DOI:** 10.1038/s41398-021-01266-1

**Published:** 2021-02-26

**Authors:** Irina Balan, Laure Aurelian, Riana Schleicher, Giorgia Boero, Todd O’Buckley, A. Leslie Morrow

**Affiliations:** 1 Department of Psychiatry, Department of Pharmacology, Bowles Center for Alcohol Studies, University of North Carolina at Chapel Hill, School of Medicine, Chapel Hill, NC 27599 USA; 2 Stanford University School of Medicine, Stanford, CA 94305 USA

**Keywords:** Physiology, Pharmacology

## Abstract

We have shown that endogenous neurosteroids, including pregnenolone and 3α,5α-THP inhibit toll-like receptor 4 (TLR4) signal activation in mouse macrophages and the brain of alcohol-preferring (P) rat, which exhibits innate TLR4 signal activation. The current studies were designed to examine whether other activated TLR signals are similarly inhibited by 3α,5α-THP. We report that 3α,5α-THP inhibits selective agonist-mediated activation of TLR2 and TLR7, but not TLR3 signaling in the RAW246.7 macrophage cell line. The TLR4 and TLR7 signals are innately activated in the amygdala and NAc from P rat brains and inhibited by 3α,5α-THP. The TLR2 and TLR3 signals are not activated in P rat brain and they are not affected by 3α,5α-THP. Co-immunoprecipitation studies indicate that 3α,5α-THP inhibits the binding of MyD88 with TLR4 or TLR7 in P rat brain, but the levels of TLR4 co-precipitating with TRIF are not altered by 3α,5α-THP treatment. Collectively, the data indicate that 3α,5α-THP inhibits MyD88- but not TRIF-dependent TLR signal activation and the production of pro-inflammatory mediators through its ability to block TLR-MyD88 binding. These results have applicability to many conditions involving pro-inflammatory TLR activation of cytokines, chemokines, and interferons and support the use of 3α,5α-THP as a therapeutic for inflammatory disease.

## Introduction

Toll-like receptors (TLRs) are a family of pattern recognition receptors that are expressed in macrophages, microglia, astrocytes, and neurons and contribute to pro-inflammatory, innate, and neuroimmune responses. They are located on the cell surface (TLR1, 2, 4, 5, 6, 11, 12) or endosomes (TLR3, 7, 8, 9, 13) and recognize molecular signatures that initiate signaling pathways for cytokine and chemokine expression, which depend on the binding of the adaptor proteins myeloid differentiation primary response 88 (MyD88) or TIR-domain-containing adapter-inducing interferon-β (TRIF)^1–6^. Most TLRs (except TLR3 and 4) signal exclusively through MyD88-dependent pathways to produce pro-inflammatory cytokines and chemokines or activate interferon pathways. TLR3 signals exclusively through TRIF-dependent pathways and TLR4 signals through both MyD88- and TRIF-dependent pathways. TLR signal activation and excessive cytokine production are implicated in the etiology of depression^7,8^, alcohol use disorders and other addictions^9,10^, traumatic brain injury^11^, neurodegeneration^3,12,13^, ischemia^14^, epilepsy^15,16^, and various systemic inflammatory conditions, including sepsis and Covid-19^17,18^.

Neurosteroid allopregnanolone ((3α,5α)3-hydroxypregnan-20-one, 3α,5α-THP) is synthesized in adrenals, gonads, and brain from cholesterol or sterol precursors. It acts upon synaptic and extrasynaptic γ-aminobutyric acid type A (GABA_A_) receptors to mediate phasic and tonic inhibition^19,20^, has anesthetic, anticonvulsant, sedative, and anxiolytic effects^21^, and modulates the hypothalamic pituitary adrenal axis to reduce stress activation^22^. Significantly, 3α,5α-THP and/or its precursors progesterone and pregnenolone, were shown to be effective in clinical studies of traumatic brain injury^23^, schizophrenia^24^, cocaine craving^25^, and post-partum depression^26^, identifying them as promising therapeutics^27,28^. Remarkably, these diverse conditions all exhibit pro-inflammatory immune and neuroimmune activation.

Recent studies showed that 3α,5α-THP, progesterone, and pregnenolone inhibit TLR4 signaling and its cognate pro-inflammatory factors, including monocyte chemoattractant protein-1 (MCP-1), high mobility group box 1 (HMGB1), and tumor necrosis factor alpha (TNF-α) in RAW264.7 cells and brain^29–31^. We showed that 3α,5α-THP inhibits the TLR activation mechanisms involving TLR4 binding to myeloid differentiation factor 2 (MD2), MyD88, and GABA_A_ α2 subunits^31^. Here, we report that 3α,5α-THP inhibits TLR activation via MyD88- but not TRIF-dependent signals to regulate a broad spectrum of pro-inflammatory responses in the innate immune system and the brain.

## Materials and methods

### Cells and reagents

Mouse macrophage/monocyte RAW264.7 cells were obtained from American Type Culture Collection (Manassas, VA, USA) and grown as previously described^31^ and details are provided in Supplemental Information (SI) Materials and Methods. The selective agonist for TLR2 [Pam_3_Cys-Ser-(Lys)_4_ hydrochloride (Pam3Cys; 10 μg/ml)] (Cat. #506350, Sigma-Aldrich, Saint Louis, MO, USA) was added to the cultures alone, or together with 3α,5α-THP (1.0 μM) in DMEM (without FBS and antibiotics) for 30 min and cells were harvested after 24 h. Selective agonists for TLR3 [polyinosinic–polycytidylic acid potassium salt (Poly(I:C); 25 μg/ml)] (Cat. #P9582, Sigma-Aldrich) or TLR7 [imiquimod (IMQ); 3 μg/ml] (Cat. #tlrl-imqs, InvivoGen, San Diego, CA, USA) were added to the cultures alone, or together with 3α,5α-THP (1.0 μM) in DMEM (without FBS and antibiotics) 24 h before cell collection.

### Antibodies

Antibodies were commercially obtained, validated and used according to the manufacturer’s instructions. Primary antibodies, their host species, clonality and dilution are listed in Table S1. Horseradish peroxidase-labeled secondary antibodies were anti-rabbit (Cat. # 7074, RRID: AB_2099233, Cell Signaling Technology), anti-mouse (Cat# 7076, RRID: AB_330924, Cell Signaling Technology), and anti-goat IgG (Cat# A24452, RRID: AB_2535921, Thermo Fisher Scientific, Waltham, MA, USA).

### Animals

Male and female alcohol-preferring (P) (males: *N* = 38; females: *N* = 38) and non-preferring (NP) rats (males: *N* = 6; females: *N* = 6) (3–4 months old; 250–550 g) were obtained from the Alcohol Research Center, Indiana University School of Medicine, double housed in Plexiglas cages, given food and water ad libitum and maintained on a 12 h light-dark cycle (light onset at 0700 h). We examined 3α,5α-THP regulation of TLR signaling in alcohol-P rats because the selective breeding of these rats for alcohol preference resulted in innate activation of TLR4 and nuclear factor kappa-light-chain-enhancer of activated B cells (NF-kB) signals^9,32–36^ as well as numerous pathological behavioral properties, including anxiety-like behavior^37,38^, impulsivity^33^, and stress reactivity^39^. Procedures followed NIH Guidelines under Institutional Animal Care and Use Committee approved protocols at the University of North Carolina, School of Medicine. All studies were conducted in the morning to avoid potential circadian fluctuations in neurosteroids^40,41^. P rats were randomly given 3α,5α-THP (15 mg/kg) (males: *N* = 16; females: *N* = 16) or vehicle (45% w/v 2-hydroxypropyl-β-cyclodextrin) (males: *N* = 16; females: *N* = 16) by intraperitoneal (IP) injection and sacrificed 30 min later. Collected brains were stored at −80 °C and nucleus accumbens (NAc) and amygdala were dissected on ice using a brain matrix. This dose and time point were selected based on previous studies showing that 3α,5α-THP has anxiolytic^42^ and anticonvulsant^43^, but not hypnotic effects^44^, and we have previously demonstrated that it inhibits TLR4 pathway activation^31^.

### Immunoblotting and co-immunoprecipitation

Protein extraction and assay were as previously described^31,32^ and details are provided in SI, Materials and Methods. Results are expressed as mean β-actin-adjusted densitometric units ± SEM or mean MyD88- or TRIF-adjusted densitometric units ± SEM.

### ELISA

Protein extraction was identical to that for immunoblotting and detailed in SI, Materials and Methods. Protein extracts were assayed with ELISA kits (Raybiotech, Norcross, GA, USA) for MCP-1 (Cat. #ERC-MCP-1-CL), TNF-α (Cat. #ELM-TNFa-CL-1), or pIRF3 (Cat. #PEL-IRF3-S386) as per the manufacturer’s instructions. Results are expressed as picograms/milligram total protein (pg/mg) or relative units/milligram total protein (RU/mg).

### Statistics

RAW264.7 cells treated with TLR agonists with/without 3α,5α-THP (*n* = 5–10/grp) were analyzed using one-way analysis of variance (ANOVA) followed by Tukey’s post hoc test. Effects of 3α,5α-THP on RAW264.7 cells (*n* = 6/grp) that were not treated with TLR agonists, were analyzed by *t*-test. Four separate cohorts of animals were used. Brain tissues (*n* = 8/grp) from male and female P rats that were given 3α,5α-THP or vehicle (Cohort 1) were analyzed using two-way ANOVA followed by Tukey’s post hoc test. Brain tissues (*n* = 8/grp) from female P rats that were given 3α,5α-THP or vehicle (Cohort 2), co-immunoprecipitation (*n* = 3–4/grp) (Cohort 2 and 3) and comparison of naïve untreated NP (*n* = 6/grp) vs. P (*n* = 6/grp) rat brain tissues (Cohort 4), were analyzed by *t*-test using Graphpad Prism 8.3.1. *P* < 0.05 was considered statistically significant.

## Results

### 3α,5α-THP inhibits the activation of MyD88-dependent TLR pathways in RAW264.7 cells

We have previously shown that 3α,5α-THP inhibits the activated TLR4 signaling pathway in cultured cells, but it has no effect on the steady state non-activated pathway^31^. Inhibition is independent of the cell type and the activating agonist. It was seen in both lipopolysaccharide (LPS)-treated RAW246.7 macrophage/monocyte cells and neuronal N2a cells, where the activator is the GABA_A_ receptor α2 subunit^31,45^. Inhibition levels were similar for 3α,5α-THP at 0.5 and 1.0 µM^31^. Pathway inhibition included blocking of the phosphorylation (activation) of canonical signaling members including transforming growth factor beta-activated kinase 1 (TAK1), NF-kB p65, and cAMP-response element-binding protein (CREB) and the resulting expression of MCP-1^31^.

To examine whether 3α,5α-THP also inhibits activation of other TLR signals and pathway members, we first studied its effect in RAW246.7 cells, an established cell line model of adaptive and innate immunity^46,47^, using selective TLR2, 3 and 7 ligands. We focused on TLRs located on the plasma membrane (TLR2) or endosomes (TLR7, TLR3) that signal exclusively through MyD88 (TLR2, TLR7) or TRIF (TLR3) binding^2,48–52^. Cells were treated with the TLR2 agonist Pam3Cys (10 µg/ml, 30 min and cells harvested after 24 h), the TLR7 agonist IMQ (3 µg/ml; 24 h) or the TLR3 agonist Poly(I:C) (25 µg/ml; 24 h), in the absence or presence of 3α,5α-THP (1.0 µM). Cell extracts were assayed for canonical members of activated pathways, including tumor necrosis factor receptor-associated factor 6 (TRAF6), activated (phosphorylated) extracellular signal-regulated kinase 1/2 (pERK1/2), pCREB, activating transcription factor 2 (pATF2), the TLR7-associated activated (phosphorylated) transcription factor interferon regulatory factor 7 (pIRF7), the TLR3-associated activated transcription factor IRF3 (pIRF3) and the cytokines/chemokines TNF-α and interferon gamma-induced protein 10 (IP-10; also known as CXCL10)^1,53–55^. The ligands and 3α,5α-THP concentrations were selected based on previous findings of maximal effects^31,56–58^. The effect of 3α,5α-THP on cells that were not treated with the TLR agonists was studied in parallel.

**Fig. 1:**
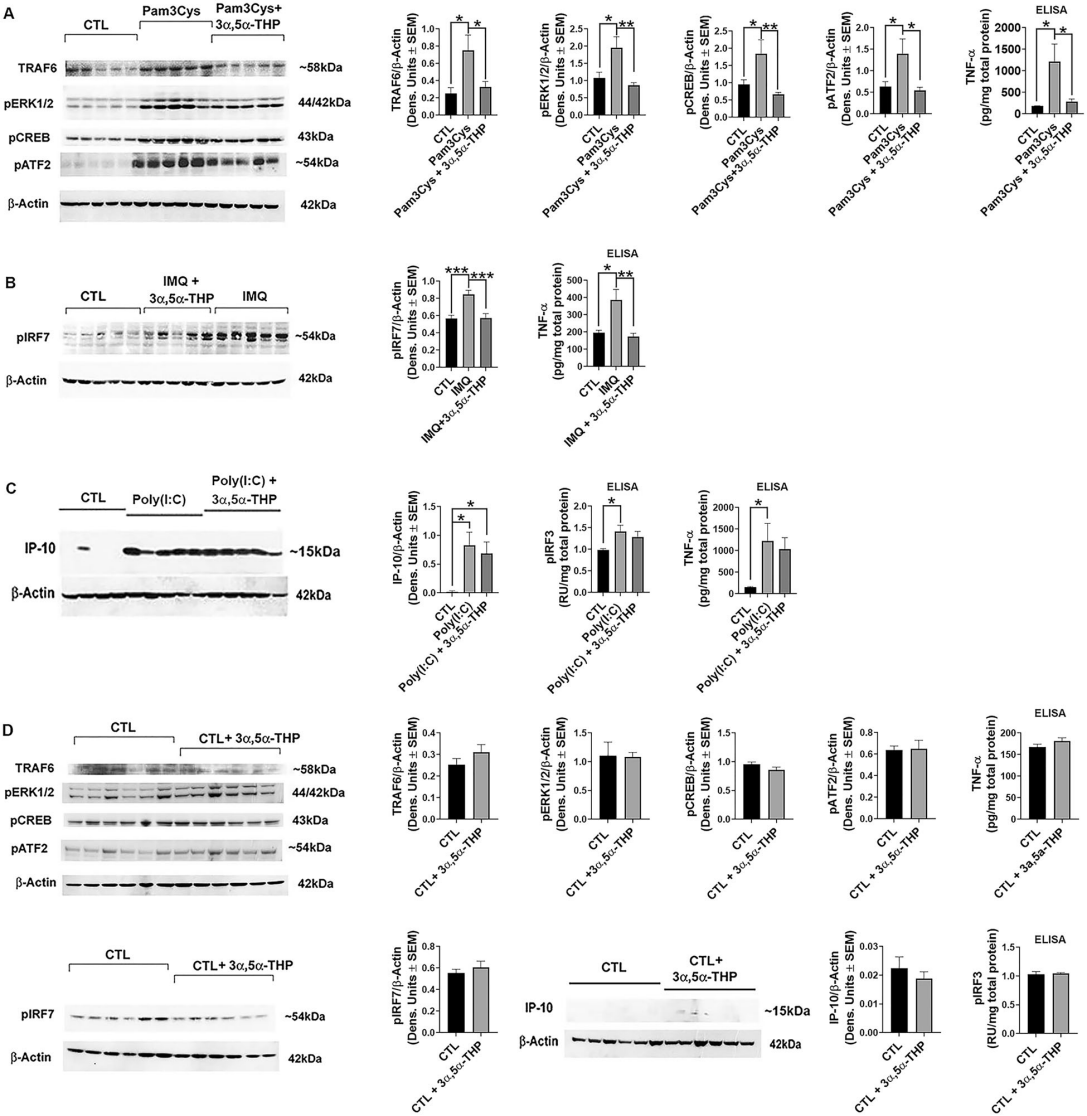
3α,5α-THP inhibits the activation of MyD88-dependent TLR pathways in RAW264.7 cells. RAW264.7 cells (*n* = 5–10/grp) were treated with Pam3Cys (10 µg/ml; 30 min) (**A**), imiquimod (IMQ; 3 µg/ml; 24 h) (**B**), or Poly(I:C) (25 µg/ml; 24 h) (**C**) with or without 3α,5α-THP (1 µM). Cells were harvested at 24 h after treatment initiation and examined for the expression of MyD88-dependent (**A**, **B**) and TRIF-dependent (**C**) signal activation. **A** Pam3Cys caused a significant increase in the levels of TRAF6 (*n* = 8/grp), pERK1/2 (*n* = 10/grp), pCREB (*n* = 10/grp), pATF2 (*n* = 10/grp), and TNF-α (*n* = 5/grp) relative to vehicle control (CTL), and these increases were completely blocked by 3α,5α-THP (One-way ANOVA, Tukey’s post hoc test: *p < 0.05; **p < 0.01). **B** IMQ caused a significant increase in the levels of pIRF7 (*n* = 9/grp) and TNF-α (*n* = 5/grp) relative to CTL, and these increases were completely blocked by 3α,5α-THP (One-way ANOVA, Tukey’s post hoc test: *p < 0.05, **p < 0.01, ***p < 0.001). **C** Poly(I:C) significantly increased the levels of pIRF3 (*n* = 5/grp), IP-10 (*n* = 6/grp), and TNF-α (*n* = 5/grp) relative to CTL (One-way ANOVA, Tukey’s post hoc test: *p < 0.05). The increases of pIRF3, IP-10, and TNF-α were not inhibited by 3α,5α-THP (One-way ANOVA, Tukey’s post hoc test: *p* > 0.05). **D** 3α,5α-THP does not target the non-activated TLR signals. RAW264.7 cells untreated with TLR agonist but exposed to vehicle or treated with 3α,5α-THP (1 µM) were harvested after 24 h. The levels of TRAF6 (*n* = 6/grp), pERK1/2 (*n* = 6/grp), pCREB (*n* = 6/grp), pATF2 (*n* = 6/grp), TNF-α (*n* = 6/grp), pIRF7 (*n* = 6/grp), IP-10 (*n* = 6/grp), and pIRF3 (*n* = 6/grp) were similar in the 3α,5α-THP-treated and untreated cells (*t*-test, *p* > 0.05). The data indicate that 3α,5α-THP specifically targets only the activated TLR signal.

Pam3Cys caused a significant increase in the levels of TRAF6 (66.3 ± 9.7%; one-way ANOVA, Tukey’s post hoc test: *p* = 0.0158, *n* = 8/grp), pERK1/2 (44.6 ± 11.9%; one-way ANOVA, Tukey’s post hoc test: *p* = 0.0191, *n* = 10/grp), pCREB (48.2 ± 12.2%; one-way ANOVA, Tukey’s post hoc test: *p* = 0.0426, *n* = 10/grp), pATF2 (54.4 ± 10.1%; one-way ANOVA, Tukey’s post hoc test: *p* = 0.0443, *n* = 10/grp) and TNF-α (84.8 ± 9.3%; one-way ANOVA, Tukey’s post hoc test: *p* = 0.0250, *n* = 5/grp) relative to untreated (CTL) cells. 3α,5α-THP inhibited the Pam3Cys effect on TRAF6 by 56.5 ± 8.7% (One-way ANOVA: *F*(2,21) = 5.453, *p* = 0.0124, *n* = 8/grp), pERK1/2 by 55.3 ± 3.7% (One-way ANOVA: *F*(2,27) = 7.314, *p* = 0.0029, *n* = 10/grp), pCREB by 63.8 ± 3.1% (One-way ANOVA: *F*(2,27) = 6.207, *p* = 0.0061, *n* = 10/grp), pATF2 by 60.9 ± 5.1% (One-way ANOVA: *F*(2,27) = 4.854, *p* = 0.0158, *n* = 10/grp) and TNF-α by 76.2 ± 4.7% (One-way ANOVA: *F*(2,12) = 5.667, *p* = 0.0185, *n* = 5/grp) (Fig. 1a). The TLR7 agonist IMQ increased the levels of pIRF7 by 33.4 ± 4.6% (One-way ANOVA, Tukey’s post hoc test: *p* = 0.0006, *n* = 9/grp) and TNF-α by 49.3 ± 9.2% (One-way ANOVA, Tukey’s post hoc test: *p* = 0.0103, *n* = 5/grp) relative to CTL cells. 3α,5α-THP inhibited the increase in pIRF7 by 32.6 ± 6.0% (One-way ANOVA: *F*(2,24) = 12.40, *p* = 0.0002, *n* = 9/grp) and the increase in TNF-α by 55.0 ± 5.1% (One-way ANOVA: *F*(2,12) = 9.520, *p* = 0.0033, *n* = 5/grp), respectively (Fig. 1b).

The levels of pIRF3, IP-10, and TNF-α were also significantly increased in cells treated with the TLR3 agonist Poly(I:C) [pIRF3: 30.7 ± 7.3% (One-way ANOVA, Tukey’s post hoc test: *p* = 0.0391, *n* = 5/grp), IP-10: 97.3 ± 0.6% (One-way ANOVA, Tukey’s post hoc test: *p* = 0.0142, *n* = 6/grp) and TNF-α: 87.7 ± 7.3% (One-way ANOVA, Tukey’s post hoc test: *p* = 0.0471, *n* = 5/grp), respectively], but these effects were not inhibited by 3α,5α-THP (One-way ANOVA, Tukey’s post hoc test: *p* = 0.6881, *p* = 0.8291, and *p* = 0.8892 for pIRF3 (*n* = 5/grp), IP-10 (*n* = 6/grp), and TNF-α (*n* = 5/grp), respectively) (Fig. 1c). Consistent with our previous findings^31^, 3α,5α-THP did not alter (*t*-test, *p* > 0.05) the expression of any pathway member in cells that were not treated with TLR agonists (Fig. 1d) indicating that 3α,5α-THP specifically targets only the activated TLR signals. Collectively, the data indicate that 3α,5α-THP inhibits the activation of both cell surface and endosomal TLR signals, but only if they are MyD88-dependent.

### 3α,5α-THP inhibits the innately activated TLR4 and TLR7 signals in the P rat brain

Neuroimmune signaling in the brain is now well established as playing an important role in brain function and it involves cross talk between multiple cell types including neurons, glia, and endothelial cells^10,59–61^. We have previously shown that 3α,5α-THP inhibits the innately activated TLR4 signal in the brain of male alcohol-P rats (ventral tegmental area)^31^. However, its effect on TLR4 signaling in female P rats, the potential existence of other innately activated TLR signaling pathways in male and female P rats, and their inhibition by 3α,5α-THP, remained unclear.

**Fig. 2:**
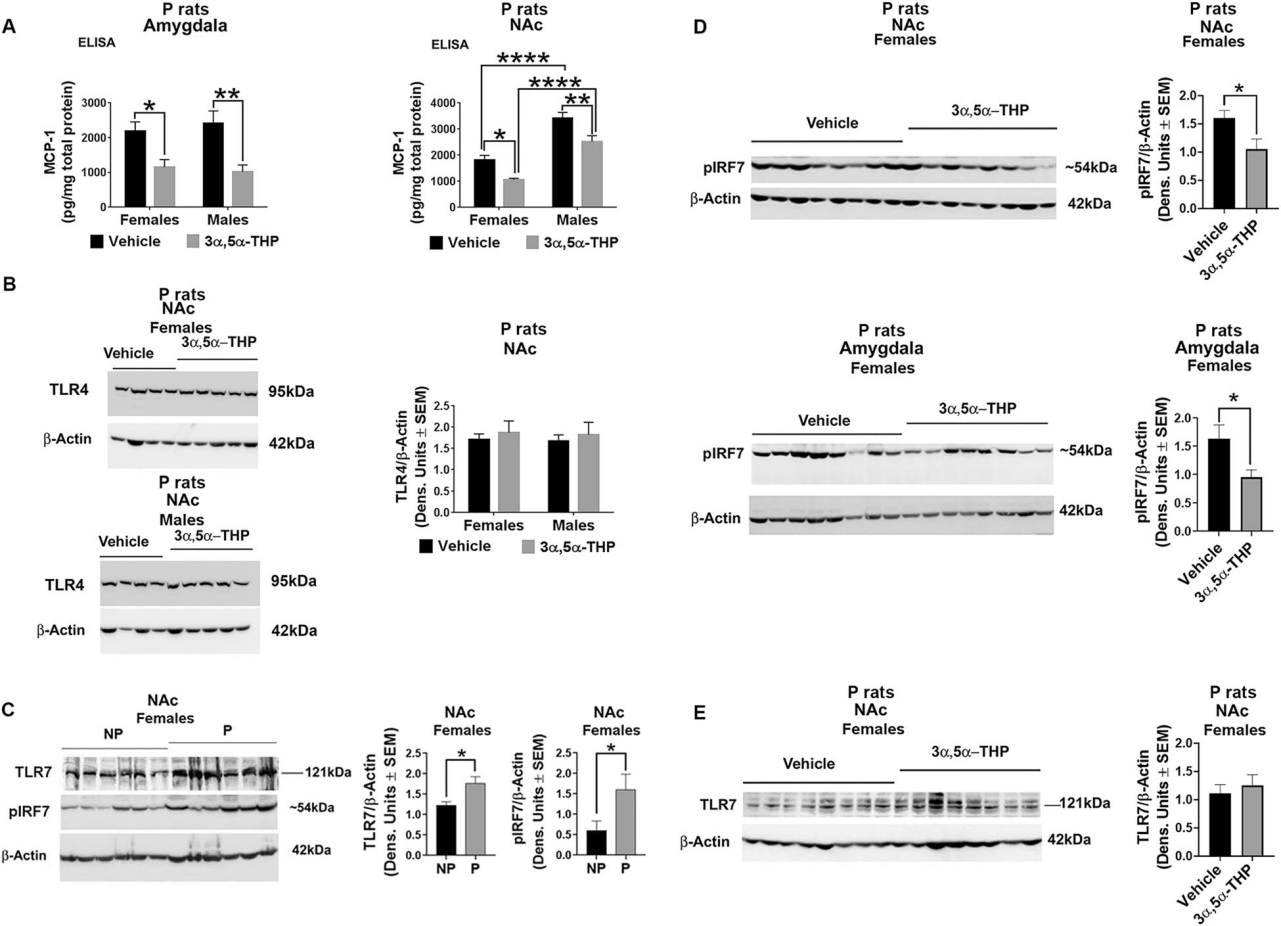
3α,5α-THP inhibits the innately activated MyD88-dependent TLR4 and TLR7 signals in the nucleus accumbens (NAc) and amygdala of P rats. **A**, **B** Male and female alcohol-preferring (P) rats (*n* = 8/grp) were treated (IP; 30 min) with 3α,5α-THP (15 mg/kg) or vehicle (45% w/v 2-hydroxypropyl-β-cyclodextrin) control and the amygdala and NAc were examined for MCP-1 and TLR4 expression. **A** There is no a significant sex difference for MCP-1 expression in the amygdala (Two-way ANOVA: *F*(1,28) = 0.02030, *p* = 0.8877, *n* = 8/grp). However, in the NAc there is a significant sex difference in the MCP-1 level (Two-way ANOVA: *F*(1,28) = 72.27, *p* < 0.0001, *n* = 8/grp). Specifically, baseline MCP-1 levels are significantly higher in the NAc from males than females (Two-way ANOVA, Tukey’s post hoc test: ****p < 0.0001, *n* = 8/grp). 3α,5α-THP administration significantly reduces MCP-1 expression in the male and female amygdala (*n* = 8/grp) and NAc (*n* = 8/grp) (Two-way ANOVA, Tukey’s post hoc test: *p < 0.05, **p < 0.01). However, the total values of MCP-1 in the presence of 3α,5α-THP are also higher in males than females (Two-way ANOVA, Tukey’s post hoc test: ****p < 0.0001, *n* = 8/grp) in P rat NAc. **B** The levels of TLR4 are similar in the males and females NAc tissues (Two-way ANOVA: *F*(1,28) = 0.06052, *p* = 0.8075, *n* = 8/grp) and they are not altered by 3α,5α-THP treatment (Two-way ANOVA: *F*(1,28) = 0.4218, *p* = 0.5213, *n* = 8/grp). **C** The TLR7 signal is innately activated in female P rats. NAc tissues from male and female alcohol-preferring (P) (*n* = 6/grp) and non-preferring (NP) control (*n* = 6/grp) rats were assayed for the expression of TLR7 and pIRF7, which is indicative of TLR7 signal activation. The levels of TLR7 and pIRF7 are significantly higher in female P than NP rats (*t*-test: *p < 0.05, *n* = 8/grp) consistent with innate TLR7 activation in female P rats. The levels of TLR7 and pIRF7 are similar in male P and NP rats (SI, Fig. S1). **D**, **E** Female P rats (*n* = 8/grp) were treated (IP; 30 min) with 3α,5α-THP or vehicle control and the NAc and amygdala were examined for expression of TLR7 and pIRF7 (indicative of TLR7 activation). 3α,5α-THP administration significantly reduced pIRF7 expression in the NAc and amygdala from female P rats (*t*-test: *p < 0.05, *n* = 8/grp) (**D**), but it had no effect on TLR7 expression in female P rat NAc (*t*-test, *p* > 0.05, *n* = 8/grp) (**E**).

Two series of experiments were done in order to address these questions. In a first series of experiments we examined the effect of 3α,5α-THP on the innately activated TLR4 signal in male and female P rats. Male and female P rats (*n* = 8/grp) were treated (30 min) with 3α,5α-THP (15 mg/kg) or vehicle control (45% w/v 2-hydroxypropyl-β-cyclodextrin) as previously described^31^ and the expression of MCP-1, [indicative of TLR4 activation^35,59^] was assessed in the amygdala and NAc. Its effect on TLR4 was studied in parallel. These brain sites were chosen based on our previous findings that the TLR4 signal is innately activated in the P rats NAc and the central nucleus of the amygdala, where it is involved in binge drinking^32,34,35,59^. 3α,5α-THP inhibited MCP-1 expression both in the amygdala (Two-way ANOVA: *F*(1,28) = 20.92, *p* < 0.0001, *n* = 8/grp) and NAc (Two-way ANOVA: *F*(1,28) = 21.14, *p* < 0.0001, *n* = 8/grp). The reduction of MCP-1 was similar in males (57.7 ± 8.2% by two-way ANOVA, Tukey’s post hoc test: *p* = 0.0049, *n* = 8/grp) and females (47.3 ± 9.7% by Two-way ANOVA, Tukey’s post hoc test: *p* = 0.046, *n* = 8/grp) in the amygdala. In the NAc, by contrast, inhibition was only 26.2 ± 6.4% (Two-way ANOVA, Tukey’s post hoc test: *p* = 0.0077, *n* = 8/grp) in males compared to 41.7 ± 3.0% (Two-way ANOVA, Tukey’s post hoc test: *p* = 0.0282, *n* = 8/grp) in females. In the amygdala, the levels of MCP-1 were similar in males and females (Two-way ANOVA: *F*(1,28) = 0.02030, *p* = 0.8877, *n* = 8/grp) but in the NAc, the MCP-1 levels were significantly higher in males than females (Two-way ANOVA: *F*(1,28) = 72.27, *p* < 0.0001, *n* = 8/grp). Specifically, the total levels of MCP-1 in the NAc were significantly higher in males than females both in the absence of 3α,5α-THP (46.7 ± 4.1% by Two-way ANOVA, Tukey’s post hoc test: *p* < 0.0001, *n* = 8/grp) and in its presence (57.9 ± 5.2% by Two-way ANOVA, Tukey’s post hoc test: *p* < 0.0001, *n* = 8/grp), identifying the NAc as a brain site with sex differences for TLR4 activation (Fig. 2a). Consistent with our previous findings^31^, the levels of TLR4 were not altered by 3α,5α-THP (Two-way ANOVA: *F*(1,28) = 0.4218, *p* = 0.5213, *n* = 8/grp) and they were similar in male and female NAc tissues (Two-way ANOVA: *F*(1,28) = 0.06052, *p* = 0.8075, *n* = 8/grp) (Fig. 2b). Collectively, the data indicate that 3α,5α-THP inhibits TLR4 signal activation in both males and females, but the activation levels are significantly higher in the NAc from males than females, even in the presence of 3α,5α-THP.

In the second series of experiments, we asked whether TLR signaling pathways other than TLR4 are also innately activated in the NAc from P but not NP rats, and whether these are inhibited by 3α,5α-THP. NAc tissues from male and female P (*n* = 6/grp) and NP (*n* = 6/grp) rats were assayed for the expression of TLR7 and pIRF7, which is indicative of TLR7 signal activation^1,53^. The levels of TLR7 (30.4 ± 7.7% by *t*-test: *t* = 2.827, df = 10, *p* = 0.0179, *n* = 6/grp) and pIRF7 (61.8 ± 18.3% by *t*-test: *t* = 2.240, df = 10, *p* = 0.0490, *n* = 6/grp) were significantly higher in female P than NP rats, consistent with innate TLR7 activation in female P rats (Fig. 2c). However, in males, the levels of TLR7 (*t*-test: *t* = 0.5318, df = 10, *p* = 0.6065, *n* = 6/grp) and pIRF7 (*t*-test: *t* = 0.3349, df = 10, *p* = 0.7446, *n* = 6/grp) were the same in P and NP rats (Fig. S1).

To examine whether 3α,5α-THP inhibits TLR7 signal activation we studied female P rats that were treated (30 min) with 3α,5α-THP (15 mg/kg) or vehicle control (45% w/v 2-hydroxypropyl-β-cyclodextrin). We found that in the NAc from female P rats, pIRF7 expression was inhibited by 3α,5α-THP by 34.1 ± 11.1% (*t*-test: *t* = 2.417, df = 14, *p* = 0.0299, *n* = 8/grp) (Fig. 2d) but the levels of TLR7 were not altered by 3α,5α-THP (*t*-test: *t* = 0.7286, df = 14, *p* = 0.4782, *n* = 8/grp) (Fig. 2e) indicating that 3α,5α-THP inhibits TLR7 signal activation but not protein expression. 3α,5α-THP also inhibited pIRF7 expression in the female P rat amygdala by 41.5 ± 8.0% (*t*-test: *t* = 2.423, df = 14, *p* = 0.0296, *n* = 8/grp). In contrast, we found no indication of innate activation of TLR2 and TLR3 signals in P rat brain (Fig. S2A, B) and 3α,5α-THP had no effect on pIRF3 in P rat NAc (Fig. S2C). The data support the conclusion that 3α,5α-THP inhibits the MyD88-dependent TLR signals, also in the brain.

### 3α,5α-THP inhibits MyD88, but not TRIF binding to TLRs

**Fig. 3:**
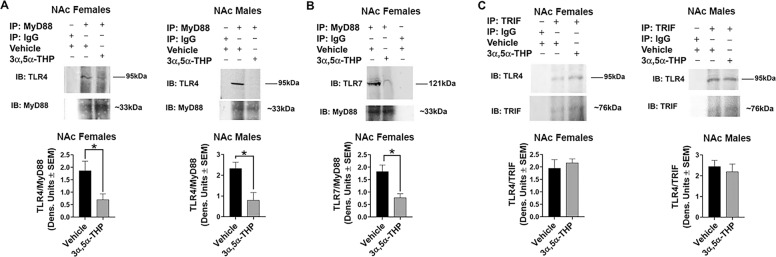
3α,5α-THP inhibits MyD88, but not TRIF binding to TLRs. Protein extracts obtained from the NAc of male and female P rats (*n* = 3–4/grp) after administration of 3α,5α-THP (15 mg/kg) or vehicle (45% w/v 2-hydroxypropyl-β-cyclodextrin) were immunoprecipitated (IP) with antibody to MyD88 (**A**, **B**) or TRIF (**C**). Proteins immunoprecipitated with MyD88 antibody were analyzed by immunoblotting (IB) with TLR4 and MyD88 antibodies (**A**) and TLR7 and MyD88 antibodies (**B**). Proteins immunoprecipitated with TRIF antibody were analyzed by immunoblotting with TLR4 and TRIF antibodies (**C**). Normal IgG was used as immunoprecipitation control. MyD88 co-precipitated with TLR4 (**A**) and TLR7 (**B**), and TRIF co-precipitated with TLR4 (**C**) but MyD88 and TRIF did not co-precipitate with normal IgG (**A**–**C**). **A** The levels of TLR4 co-precipitating with MyD88 were significantly reduced by 3α,5α-THP both in males (*n* = 3/grp) and females (*n* = 4/grp) (*t*-test, *p < 0.05). **B** The levels of TLR7 co-precipitating with MyD88 were also significantly reduced by 3α,5α-THP in females (*t*-test, *p < 0.05, *n* = 3/grp). **C** The levels of TLR4 co-precipitating with TRIF were not altered by 3α,5α-THP treatment both in males (*n* = 3/grp) and females (*n* = 3/grp) (*t*-test, *p* > 0.05).

To further confirm that 3α,5α-THP inhibits MyD88- but not TRIF- dependent signals and examine the potential mechanism of inhibition, protein extracts from the NAc of male and female P rats treated with 3α,5α-THP or vehicle control (*n* = 3–4/grp) were immunoprecipitated with antibody to MyD88 or TRIF. The MyD88 and TRIF precipitates from both males and females were immunoblotted with antibody to TLR4 and the MyD88 precipitates from females were immunoblotted with antibody to TLR7. Immunoblotting with MyD88 or TRIF antibodies served to confirm immunoprecipitation and normal IgG was used as immunoprecipitating antibody control. We found that MyD88 co-precipitates with TLR4 and TLR7 and TRIF co-precipitates with TLR4. The levels of TLR4 co-precipitating with MyD88 were significantly reduced by 3α,5α-THP in both males (65.9 ± 15.9% by *t*-test: *t* = 3.237, df = 4, *p* = 0.0318, *n* = 3/grp) and females (62.3 ± 12.4% by *t*-test: *t* = 2.596, df = 6, *p* = 0.0409, *n* = 4/grp). 3α,5α-THP also inhibited the levels of TLR7 co-precipitating with MyD88 in females (57.7 ± 8.8% by *t*-test: *t* = 3.500, df = 4, *p* = 0.0249, *n* = 3/grp) (Fig. 3a, b), but it did not alter the levels of TLR4/TRIF binding both in males (*t*-test: *t* = 0.5593, df = 4, *p* = 0.6058, *n* = 3/grp) or females (*t*-test: *t* = 0.5682, df = 4, *p* = 0.6003, *n* = 3/grp) (Fig. 3c). Collectively, the data indicate that 3α,5α-THP inhibits MyD88, but not TRIF binding to TLRs, thereby inhibiting the MyD88- but not the TRIF-dependent signal.

## Discussion

The salient feature of the data presented in this report is the finding that 3α,5α-THP inhibits the activation of MyD88- but not TRIF-dependent TLR signals in cultured macrophage cells and brain, and that brain site and sex appear to affect the resulting pro-inflammatory response. Since most TLRs signal through MyD88-dependent pathways, it would appear that the endogenous neurosteroid 3α,5α-THP has remarkable and heretofore unappreciated potential to prevent or ameliorate inflammatory pathway signals and possibly inflammatory conditions.

TLR signals impact infectious diseases and non-infectious CNS disorders, learning, memory, and neurogenesis^18,61,62^. The TLR4 signal contributes to various neuropsychiatric conditions, addiction^3,9,63^, and depression^8^. The TLR2 signal is involved in Alzheimer’s disease pathogenesis^64^ and multiple sclerosis brain lesions^65^. The TLR7 signal enhances contextual fear memory and depression-like behaviors in mice^66^ and the TLR3 signal is involved in anxiety-related behaviors, impairs motor coordination, and cues fear memory^12^. Both TLR4 and TLR3 are associated with alcohol consumption, albeit in distinct models and at different stages. The innately activated TLR4 signal in P rats regulates the voluntary initiation of alcohol consumption^9,34,35,59^ while Poly(I:C)-induced TLR3/TRIF-dependent signaling was seen in C57BL/6J mice maintained on an alcohol diet^67^.

TLRs share common structural domains, which define their ability to recruit the adaptor proteins that regulate signaling. All TLRs except for TLR3, signal through the MyD88 adaptor, the recruitment of which requires binding of the bridging adaptor MyD88 adaptor-like (MAL)/TIR domain-containing adaptor protein (TIRAP)^2,68^. TLR3 signals exclusively via the TRIF adaptor, and TLR4 signals through both MyD88 and TRIF^2^. TRIF recruitment requires binding of the bridging adaptor TRIF-related adaptor molecule^2,69^. Available data suggest that the TLR4/MyD88 pathway positively regulates the expression of pro-inflammatory cytokines (viz. TNF-α, IL-6, IL-1) and chemokines (viz. MCP-1)^6,35,70,71^ and microglial pro-inflammatory (M1) polarization^72^, but the TLR4/TRIF signal may be anti-inflammatory and neuroprotective^73–76^.

Since 3α,5α-THP inhibits the activated TLR4 signaling pathway in cultured cells, but it has no effect on the steady state non-activated pathway^31^, we first studied its effects on other TLRs in RAW246.7 cells using ligands specific for the activation of TLRs located on the plasma membrane (TLR2) or endosomes (TLR7, TLR3) that signal exclusively through MyD88 (TLR2, TLR7) or TRIF (TLR3) binding^2,48–52^. The ligands were used at the lowest dose that induced maximal activation responses as determined in independent studies and previously confirmed by us^31,56–58^. We found that 3α,5α-THP (1.0 µM) inhibits the activation of both cell surface and endosomal TLR signals that are MyD88-dependent, but it has no effect on the TRIF-dependent signal. Our previous data indicate that TLR inhibition levels are similar for 3α,5α-THP at 0.5 and 1.0 µM^31^. However, additional studies are needed in order to define the potency of 3α,5α-THP in inhibiting all of the TLR pathways.

**Fig. 4:**
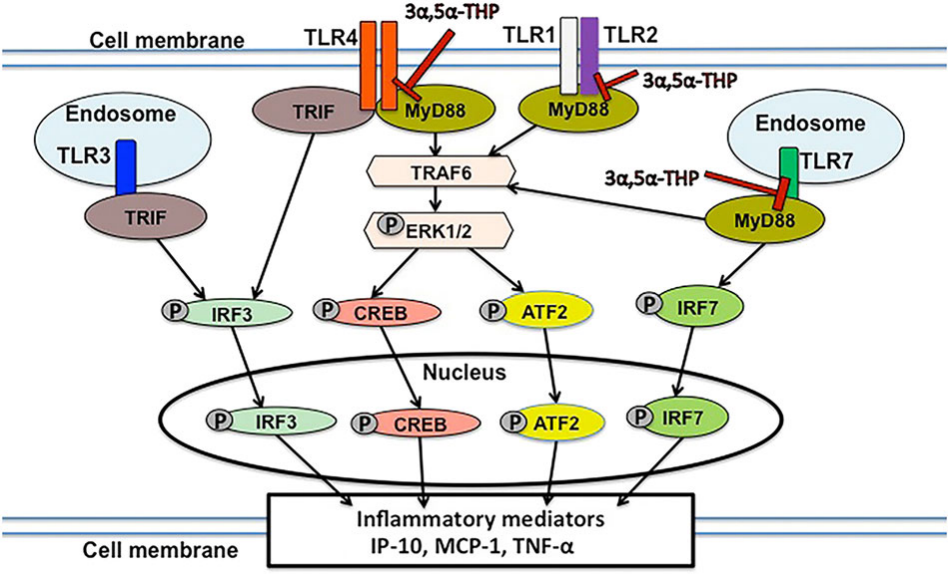
Schematic of TLR signal inhibition by 3α,5α-THP. TLR4, TLR2, and TLR7 signal activation initiates with TLR binding of MyD88, and this binding is inhibited by 3α,5α-THP. This results in the inhibition of pathway activation, including TRAF6 and activated (phosphorylated) downstream members (pERK1/2, pCREB, pATF2, and pIRF7). The activated transcription factors translocate to the nucleus and initiate the production of various inflammatory mediators (IP-10, MCP-1, and TNF-α). TLR4 also binds TRIF, which is the only adaptor protein bound by TLR3, leading to the phosphorylation (activation) of IRF3. Significantly, 3α,5α-THP does not inhibit TRIF binding and its resulting signal activation.

We found no indication of innate activation of TLR3 signals both in male and female P rat brain and TLR7 signal is activated in female but not male P rat brain as indicated by higher levels of TLR7 and pIRF7 in P vs. NP rat brains. TLR7 is encoded by an X chromosome locus and in female somatic cells there is a random silencing of one of the two X chromosomes^77,78^. However, for example, TLR7 may escape the X chromosome inactivation in immune cells^77^. The increased levels of TLR7 in female P vs. NP rat brains may be due to the reactivation of the second X chromosome controlled by non-coding RNAs, such as Xist^79^. 3α,5α-THP inhibited expression of the activated TLR7 signal pIRF7 in female P rat brain. Specifically, the reduction of pIRF7 was similar (~35–40%) in the NAc and amygdala from female P rats, indicating this effect is not isolated to NAc. Significantly, 3α,5α-THP did not inhibit pIRF3 expression (TRIF-dependent TLR3 and TLR4 signal) and co-immunoprecipitation studies confirmed that 3α,5α-THP inhibits MyD88 binding to TLR4 and TLR7, but not TRIF binding to TLR3 or TLR4 in P rat NAc lysates. Collectively, the data indicate that 3α,5α-THP inhibits MyD88, but not TRIF-dependent TLR signaling (Fig. 4).

The identity of the innate agonists that activate the TLR signals in the brain is unclear. We have shown that in neurons, the TLR4 signal is activated by binding with the α2 subunit of GABA_A_ receptors^9,34,59^, but the role of the α2 subunit in the activation of other TLR signals and in other cell types is still unclear. The differential TLR4 and TLR7 signal activation in a sex-dependent manner and/or the potential TLR-TLR cross talk and its resulting signal regulatory outcome are also not well understood. For example, the neuropeptide corticotropin-releasing factor (CRF) alters different circuits in males and females with brain region specific sensitivity^80^ and it promotes sex differences in the reinforcing effects of nicotine^81^. CRF regulates an amplification loop for the innately activated TLR4 signal that is focused on pERK1/2, downstream of TRAF6 activation^32^, a site that also applies to TLR7 signaling (Fig. 4). Also, the endogenous TLR4 agonist HMGB1 facilitates the release of the TLR7 agonist miRNA let-7b^82^ indicative of TLR4/TLR7 cross talk. This cross talk is likely not involved in signal activation in our study, because 3α,5α-THP inhibits LPS-induced HMGB1 increase^31^ and we find that in the P rat NAc, the TLR4 signal is primarily activated in males and the TLR7 signal is only activated in females, but not in males. Increased TLR4 activation in males as opposed to TLR7 in females may also reflect antagonistic effects of TLRs cross talk, as suggested by the finding that the TLR7 ligand miR-142–3p inhibits TLR4 activation and TLR4 represses miR-142–3p expression^83–85^. However, the possibility of TLR4/TLR7 cross talk in macrophages and at brain sites without sex differences cannot be excluded. Also MCP-1 is expressed in neurons, astrocytes, and microglia^35,86–88^ and it is still unclear whether the 3α,5α-THP-mediated inhibition is cell type specific. Moreover, MyD88 regulates signaling downstream of interleukin-1 receptor (IL-1R)^89,90^, but the effect of 3α,5α-THP on IL-1R/MyD88 signaling is unknown. Ongoing studies are designed to address these questions.

Our data indicate that 3α,5α-THP inhibits TLR binding of MyD88. However, the exact mechanism of the 3α,5α-THP inhibitory effect is also still unclear. One possibility is that 3α,5α-THP binds MyD88 or the bridging adaptor protein MAL/TIRAP thereby preventing MyD88 recruitment to the TLRs. 3α,5α-THP could also cause the degradation of bound MyD88, as was recently shown for pregnenolone that promotes the ubiquitination and degradation of TIRAP and TLR2 in human embryonic HEK293T cells^29^. Further studies are needed in order to detect the proteins that are bound by 3α,5α-THP, determine their localization with single-molecule resolution, and enable quantification and kinetic analysis. This is particularly relevant since inhibition of protein binding may require specific ring structures as suggested by the finding that the structure of the steroid D ring common to 3α,5α-THP, progesterone and pregnenolone, is associated with inhibition of pro-inflammatory signals^19–21,31,91^. The potential cross talk between diverse activated TLR pathways, their ability to potentially compensate for 3α,5α-THP-inhibited signals^92^ and differentially affect other cell types (viz. neurons-microglia) are additional questions in need of further elucidation.

The IP administration of 3α,5α-THP (30 min) inhibited the expression of MCP-1 (MyD88-dependent TLR signal) both in male and female P rat brain. Specifically, in the amygdala the reduction of MCP-1 was similar (~50–55%) in males and females, however in the NAc, inhibition was only ~25% in males compared to ~40% in females, while the total levels of MCP-1 in the NAc are significantly higher in males than females, both in the vehicle- and 3α,5α-THP-treated P rats. Sexually dimorphic behaviors in P rats are unknown and the significance of sex differences in TLR4 and TLR7 activation in P rat brain is unclear. However, they may be related to sex differences in ethanol consumption (F»M) as shown for Sprague–Dawley rats^93^. Future studies will use state-of-the-art mass spectroscopic methods to determine the serum or brain levels of allopregnanolone reached after treatment with allopregnanolone vs. vehicle in both males and females in order to determine whether the impact of allopregnanolone on the regulation of inflammation occurs at supra-physiological or physiological levels that may be present at rest, after stress, or in estrous phases with higher allopregnanolone levels in females.

In summary, the ability of 3α,5α-THP to inhibit MyD88-dependent TLR signals suggests a wide applicability to many inflammatory conditions that involve TLR activation of pro-inflammatory pathways^27,28,31^. Since neurosteroids, like immune factors, circulate in the bloodstream, cross the blood brain barrier, diffuse between different cell types, and exhibit paracrine effects in many cells, it is likely that neurosteroids are endogenous modulators of TLR activation and may contribute to inflammatory disease susceptibility and recovery. These results have applicability to many conditions involving pro-inflammatory TLR activation of cytokines, chemokines, and interferons.

## Supplementary information

Supplemental Material
