# Supplementary material for: Neurosteroid allopregnanolone (3α,5α-THP) inhibits inflammatory signals induced by activated MyD88-dependent toll-like receptors
Source: Transl Psychiatry. 2021 Feb 26;11:145. doi: 10.1038/s41398-021-01266-1 (PMC7909379; doi:10.1038/s41398-021-01266-1)
Supplement: Supplementary file 1 — Supplemental Material [file 41398_2021_1266_MOESM1_ESM.pdf]

SUPPLEMENTAL INFORMATION

Neurosteroid allopregnanolone ((3α,5α)3-Hydroxypregnan-20-one, 3α,5α-THP) inhibits inflammatory signals induced by activated MyD88-dependent Toll-like receptors

Irina Balan, Ph.D.[1]*, Laure Aurelian, Ph.D.[2]*, Riana Schleicher, B.S.[1], Giorgia Boero, Ph.D.[1], Todd O'Buckley, B.S[1], and A. Leslie Morrow, Ph.D.[1]

MATERIALS AND METHODS

Cells. Mouse macrophage/monocyte (RAW264.7) cells were obtained from American Type Culture Collection (Manassas, VA, USA). They were grown in Dulbecco's modified Eagle's medium (DMEM) (Gibco; Gaithersburg, MD, USA) with 10% fetal bovine serum (FBS, Gemini, West Sacramento, CA, USA), 1% penicillin/streptomycin 100× (Gibco) at 37 °C in a 5% CO2 humidified atmosphere and refreshed with media containing 2% FBS 16 hrs prior to experimentation.

Protein extraction for immunoblotting and ELISA. RAW264.7 cells were lysed with radioimmunoprecipitation (RIPA) buffer (Sigma, Cat. # R0278) supplemented with protease and phosphatase inhibitor cocktails (Sigma). Dissected NAc and amygdala tissues were lysed with CelLytic MT (dialyzable mild detergent, bicine, and 150 mM NaCl; Sigma Aldrich, St. Louis, MO, USA, Cat. # C3228) supplemented with protease and phosphatase inhibitor cocktails (Sigma). The lysates were sonicated twice for 30 seconds at 25% output power with a Sonicator ultrasonic processor (Misonix, Inc., Farmingdale, NY) and centrifuged (14,000 g; 4°C) for 30 min. Total protein levels were determined by the bicinchoninic acid assay (BCA, Thermo Fisher Scientific, Waltham, MA, USA, Cat.# 23228 and Cat.# 1859078).

Immunoblotting. The proteins (100 µg/lane) were denatured at 95°C (5 min) in 4x Laemmli denaturing buffer (Bio-Rad, Cat. # 1610747) with 10% β-mercaptoethanol and resolved by SDS-polyacrylamide gel (SDS-PAGE) electrophoresis as previously described (1-7). Briefly, the 10% separation gels (16x18cm) and 3% stacking gels were prepared from acrylamide/bisacrylamide (ratio 29:1) stock solution (Bio-Rad, Cat. # 161-0156) and were polymerized by the addition of 0.025% tetramethylethylenediamine (TEMED; Bio-Rad, Cat. # 1610800EDU) and ammonium persulphate (Bio-Rad, Cat. # 7727540). Electrophoresis was carried out with a current of 25 mA per gel for 4-5 hours. Electrophoretically separated samples were transferred to a polyvinylidene difluoride membrane (PVDF; Bio-Rad Cat. #1620177). Blots were blocked for 2 hrs at room temperature (RT) with 5% blotting-grade blocker (Bio-Rad, Cat. # 1706404) or 5% BSA (for phosphorylated primary antibodies) and exposed to primary antibodies overnight (4°C), followed by horseradish peroxidase-labeled secondary antibodies (1 hr, RT). Primary and secondary antibodies were diluted with 5% blotting-grade blocker buffer or 5% BSA (for phosphorylated primary antibodies). Tris-buffered saline with 0.05% Tween-20 (TNT) was used to wash the blots 3 times (10 min each) after incubation with primary and secondary antibodies. Immunoreactive bands were visualized with the Plus-ECL kit reagents (Perkin Elmer, Waltham, MA, USA, Cat.# NEL105001EA) followed by detection with enhanced chemiluminescence (ImageQuant LAS4000, GE Healthcare, Amersham, UK). Densitometric analysis was conducted using ImageQuant TL v8.1.0.0. Each densitometric measurement was divided by the corresponding β-actin densitometric measurement and the results are expressed as the mean β-actin-adjusted densitometric units ± SEM. The antibodies, their clonality, host species, dilution and supplier are listed in Table S1. All antibodies were validated by the supplier and by us, as previously described (2, 3, 5). Specific protein detection used full length gels (1-5, 7).

Table S1. Clonality, host species and dilutions of primary antibodies used in immunoblotting and co-immunoprecipitation

Target	Catalog no	Commercial supplier	Clonality	Host	Dilution
pERK1/2 (Thr202/Tyr204)	4377	Cell Signaling Technology, Danvers, MA, USA	Monoclonal	Rabbit	1:1000
pIRF7 (Ser437/438)	24129	Cell Signaling Technology, Danvers, MA, USA	Monoclonal	Rabbit	1:1000
pATF2 (Thr71)	9221	Cell Signaling Technology, Danvers, MA, USA	Polyclonal	Rabbit	1:1000
pCREB (Ser133)	9198	Cell Signaling Technology, Danvers, MA, USA	Monoclonal	Rabbit	1:1000
MyD88	4283	Cell Signaling Technology, Danvers, MA, USA	Monoclonal	Rabbit	1:500
TRIF	LS-C749	Lifespan Biosciences, Seattle, WA, USA	Polyclonal	Rabbit	1:200
IP-10	AF-466-NA	R&D Systems, Minneapolis, MN, USA	Polyclonal	Goat	1:200
TRAF6	sc-8409	Santa Cruz Biotechnology, Santa Cruz, CA, USA	Monoclonal	Mouse	1:200
TLR4	sc-293072	Santa Cruz Biotechnology, Santa Cruz, CA, USA	Monoclonal	Mouse	1:1000
TLR7	3269	ProSci Incorporated, Poway, CA, USA	Polyclonal	Rabbit	1:1000
TLR3	PK-AB577-3445	PromoCell, Heidelberg, Germany	Polyclonal	Rabbit	1:500
TLR2	PK-AB577-3552	PromoCell, Heidelberg, Germany	Polyclonal	Rabbit	1:500
β-Actin	66009-1-Ig	Proteintech Group, Rosemont, IL, USA	Monoclonal	Mouse	1:3000
Normal IgG	2729	Cell Signaling Technology, Danvers, MA, USA	Polyclonal	Rabbit	1:200

Co-Immunoprecipitation. NAc tissues were collected from male and female P rats treated (30 min, IP) with 3α,5α-THP (15 mg/kg) or vehicle (45% w/v 2-hydroxypropyl-β-cyclodextrin) (n=3-4/group (grp)). Proteins were extracted with CelLytic MT buffer supplemented with protease and phosphatase inhibitor cocktails and assayed for co-immunoprecipitation as previously described (5). Specifically, protein lysates (150 µg) were incubated with rabbit anti-MyD88 antibody (dilution 1:50), rabbit anti-TRIF antibody (dilution 1:50), or normal rabbit IgG (dilution 1:200) corresponding to the host species of the primary antibody and Peirce Protein A/G IgG binding buffer (up to 400 µl; Thermo Fisher Scientific, Cat. # 54200) for 1 hr at 4°C on a rocker. The mixtures were incubated with Protein A/G Plus-Agarose beads (60 µl) (Santa Cruz Biotechnology, Cat. # sc-2003) overnight at 4°C on a rocker. The immunoprecipitates were washed four times with ice-cold Pierce IP Lysis Buffer (Thermo Fisher Scientific, Cat. # 87787) by centrifugation (1000 g; 4°C) and the bound proteins were eluted at 95°C (5 min) in 2x Laemmli denaturing buffer (Bio-Rad, Cat. # 1610747) with 10% β-mercaptoethanol and resolved by SDS-polyacrylamide gel (SDS-PAGE) electrophoresis. Proteins were transferred to PVDF membranes and immunoblotted with TLR4 (dilution 1:1000), TLR7 (dilution 1:1000), MyD88 (dilution 1:500), or TRIF (dilution 1:200) antibodies. Immunoreactive bands were visualized with the Plus-ECL kit reagents (Perkin Elmer, Waltham, MA, USA, Cat.# NEL105001EA) followed by detection with enhanced chemiluminescence (ImageQuant LAS4000, GE Healthcare, Amersham, UK). Densitometric analysis was conducted using ImageQuant TL v8.1.0.0. Each densitometric measurement for TLR4 or TLR7 was divided by the corresponding MyD88 (for TLR4 or TLR7) or TRIF (for TLR4) densitometric measurement, and the results are expressed as the mean MyD88- or TRIF-adjusted densitometric units ± SEM.

RESULTS

<u>The TLR7 signal is not innately activated in the NAc from male P rats.</u> Having seen that the TLR7 signal is innately activated in the NAc from female P rats (MS, Fig.2C), we wanted to know whether it is also activated in the NAc from male P rats. NAc tissues from untreated alcohol naïve alcohol preferring (P) and non-preferring (NP) male rats (n=6/grp) were assayed for the expression of TLR7 and pIRF7. The levels of TLR7 (t-test: t=0.5318, df=10, p=0.6065, n=6/grp) and pIRF7 (t-test: t=0.3349, df=10, p=0.7446, n=6/grp) were similar in the male NP and P rats (Fig.S1). This indicates that the levels of TLR7 (30.4±7.7% by t-test: t=2.827, df=10, p=0.0179, n=6/grp) and pIRF7 (61.8±18.3% by t-test: t=2.240, df=10, p=0.0490, n=6/grp) are significantly higher in the NAc from female (but not male) P than NP rats, consistent with innate TLR7 activation in female P rats (MS, Fig. 2C).

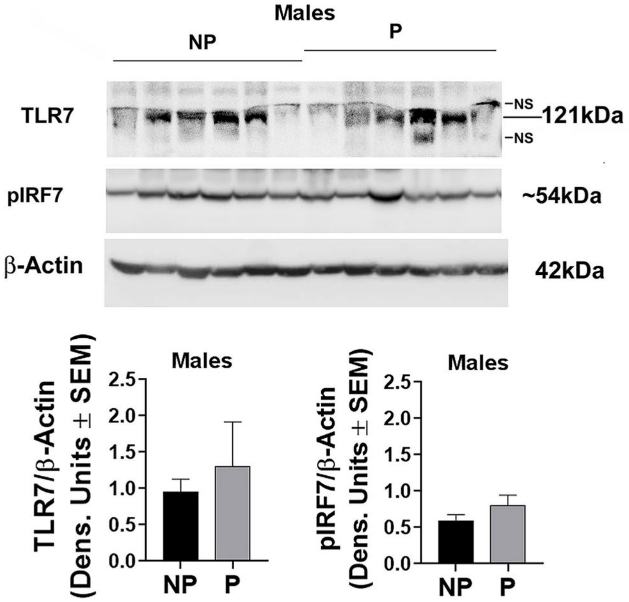

Fig. S1. The TLR7 signal is not innately activated in the NAc from male P rats. The levels of TLR7 and pIRF7 (indicative of TLR7 activation) are similar in P and NP rats.

The TLR2 and TLR3 signals are not innately activated in the NAc from male and female P rats.

NAc tissues from untreated alcohol naïve P and NP male and female rats (n=6/grp) were assayed for the expression of TLR2 and TLR3 and pIRF3. Because both TLR2 and TLR4 signal through MCP1 activation (4, 8-10), the TLR2-specific pathway member indicative of activation is still unknown. However, pIRF3 is indicative of TLR3 activation. The levels of TLR2 were similar in the NAc from male and female P and NP rats (t-test, $p>0.05$, n=6/grp) (Fig. S2A). The levels of full length TLR3 ($TLR3_{FL}$), a cleaved TLR3 sequence ($TLR3_{Cleaved}$) and pIRF3 were also similar in the NAc from male and female P and NP rats (t-test, $p>0.05$, n=6/grp). Significantly, the levels of pIRF3 are also similar (Two-way ANOVA: $F(1,28)=0.2709$, $p=0.6068$, n=8/grp) in the NAc from male and female P rats treated (30 min, IP) with 3α,5α-THP (15 mg/kg) or vehicle control (45% w/v 2-hydroxypropyl-β-cyclodextrin) (Fig. S2B). The data indicate that the TLR2 and TLR3 signals are not activated in the NAc from male and female P rats and the signal is not inhibited by 3α,5α-THP.

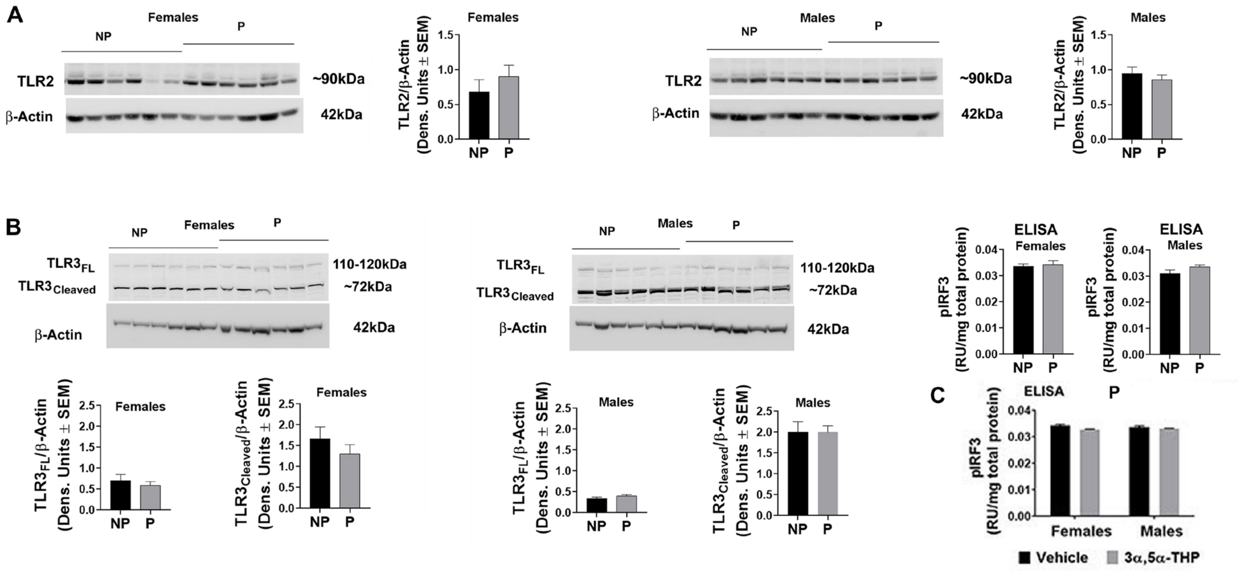

Fig. S2. The TLR2 (A) and TLR3 (B) signals are not innately activated in P rats and 3α,5α-THP had no effect on pIRF3 in the NAc from male and female P rats (C).

References.

1. Aurelian L, Warnock KT, Balan I, Puche A, June H. TLR4 signaling in VTA dopaminergic neurons regulates impulsivity through tyrosine hydroxylase modulation. Transl Psychiatry. 2016;6:e815.

2. Balan I, Warnock KT, Puche A, Gondre-Lewis MC, Aurelian L. Innately activated TLR4 signal in the nucleus accumbens is sustained by CRF amplification loop and regulates impulsivity. Brain Behav Immun. 2018;69:139-53.

3. Balan I, Warnock KT, Puche A, Gondre-Lewis MC, June H, Aurelian L. The GABAA Receptor alpha2 Subunit Activates a Neuronal TLR4 Signal in the Ventral Tegmental Area that Regulates Alcohol and Nicotine Abuse. Brain Sci. 2018;8(4).

4. June HL, Liu J, Warnock KT, Bell KA, Balan I, Bollino D, et al. CRF-amplified neuronal TLR4/MCP-1 signaling regulates alcohol self-administration. Neuropsychopharmacology. 2015;40(6):1549-59.

5. Balan I, Beattie MC, O'Buckley TK, Aurelian L, Morrow AL. Endogenous Neurosteroid (3alpha,5alpha)3-Hydroxypregnan-20-one Inhibits Toll-like-4 Receptor Activation and Pro-inflammatory Signaling in Macrophages and Brain. Sci Rep. 2019;9(1):1220.

6. Laemmli UK. Cleavage of structural proteins during the assembly of the head of bacteriophage T4. Nature. 1970;227(5259):680-5.

7. Liu J, Yang AR, Kelly T, Puche A, Esoga C, June HL, Jr., et al. Binge alcohol drinking is associated with GABAA alpha2-regulated Toll-like receptor 4 (TLR4) expression in the central amygdala. Proc Natl Acad Sci U S A. 2011;108(11):4465-70.

8. Bohacek I, Cordeau P, Lalancette-Hebert M, Gorup D, Weng YC, Gajovic S, et al. Toll-like receptor 2 deficiency leads to delayed exacerbation of ischemic injury. J Neuroinflammation. 2012;9:191.

9. Aurelian L, Balan I. GABAAR alpha2-activated neuroimmune signal controls binge drinking and impulsivity through regulation of the CCL2/CX3CL1 balance. Psychopharmacology (Berl). 2019;236(10):3023-43.

10. Du X, Fleiss B, Li H, D'Angelo B, Sun Y, Zhu C, et al. Systemic stimulation of TLR2 impairs neonatal mouse brain development. PLoS One. 2011;6(5):e19583.
